# Effects of a Metabotropic Glutamate 1 Receptor Antagonist on Light Responses of Retinal Ganglion Cells in a Rat Model of Retinitis Pigmentosa

**DOI:** 10.1371/journal.pone.0079126

**Published:** 2013-10-28

**Authors:** Ralph J. Jensen

**Affiliations:** VA Boston Healthcare System, Boston, Massachusetts, United States of America; The University of Melbourne, Australia

## Abstract

**Background:**

Retinitis pigmentosa (RP) is a progressive retinal degenerative disease that causes deterioration of rod and cone photoreceptors. A well-studied animal model of RP is the transgenic P23H rat, which carries a mutation in the rhodopsin gene. Previously, I reported that blocking retinal GABA_C_ receptors in the P23H rat increases light responsiveness of retinal ganglion cells (RGCs). Because activation of metabotropic glutamate 1 (mGlu1) receptors may enhance the release of GABA onto GABA_C_ receptors, I examined the possibility that blocking retinal mGlu1 receptors might in itself increase light responsiveness of RGCs in the P23H rat.

**Methodology/Principal Findings:**

Electrical recordings were made from RGCs in isolated P23H rat retinas. Spike activity of RGCs was measured in response to brief flashes of light over a range of light intensities. Intensity-response curves were evaluated prior to and during bath application of the mGlu1 receptor antagonist JNJ16259685. I found that JNJ16259685 increased light sensitivity of all ON-center RGCs and most OFF-center RGCs studied. RGCs that were least sensitive to light showed the greatest JNJ16259685-induced increase in light sensitivity. On average, light sensitivity increased in ON-center RGCs by 0.58 log unit and in OFF-center RGCs by 0.13 log unit. JNJ16259685 increased the maximum peak response of ON-center RGCs by 7% but had no significant effect on the maximum peak response of OFF-center RGCs. The effects of JNJ16259685 on ON-center RGCs were occluded by a GABA_C_ receptor antagonist.

**Conclusions:**

The results of this study indicate that blocking retinal mGlu1 receptors in a rodent model of human RP potentiates transmission of any, weak signals originating from photoreceptors. This augmentation of photoreceptor-mediated signals to RGCs occurs presumably through a reduction in GABA_C_-mediated inhibition.

## Introduction

Retinitis pigmentosa (RP) is an inherited form of retinal degeneration that affects approximately 1 in 4000 people worldwide. In the early stage of RP, patients have difficulty seeing at night due to loss of rod photoreceptors, and this is followed by a progressive loss of daytime vision as cone photoreceptors degenerate. Mutations in over 40 genes are known to cause RP [1]. Some of these mutations occur within the rhodopsin gene, which is expressed in rod photoreceptors. The first RP mutation identified in human patients was found to be a proline-to-histidine substitution at codon 23 (P23H) of the rhodopsin protein [2], which is a common cause of autosomal dominant RP. The present study was conducted on the transgenic P23H rat. In the P23H rat and other animal models of RP, structural changes occur in inner retinal neurons during the course of photoreceptor loss [3], [4], [5], [6], [7], [8], [9]. Abnormalities in either bipolar cells or amacrine cells may contribute to the visual deficits in RP patients.

Recently, I reported that blocking GABA_C_ receptors in the retinas of P23H rats increases light responsiveness of retinal ganglion cells (RGCs) [10]. GABA_C_ receptors are located predominately on the axon terminals of retinal bipolar cells, where they limit the release of glutamate onto RGCs [11], [12], [13], [14]. Vigh et al. [15] reported that activation of metabotropic glutamate 1 (mGlu1) receptors enhances the reciprocal GABAergic feedback to the axon terminals of Mb-type bipolar cells of the goldfish retina. In the rat retina, mGlu1 receptors may function in a similar manner: rat bipolar cells also receive reciprocal GABAergic feedback [16] and mGlu1 receptors are present in amacrine cell processes that are postsynaptic to rat bipolar cell terminals [17]. Thus, a possible alternative approach to increasing light responsiveness of RGCs in P23H rat retinas is to reduce the levels of GABA that are present at GABA_C_ receptors by antagonizing the effects of glutamate on retinal mGlu1 receptors. In the present study, I examined the effects of a mGlu1 receptor antagonist on the light responses of RGCs in the P23H rat retina.

## Materials and Methods

### Ethics Statement

This study was carried out in strict accordance with the ARVO Statement for the Use of Animals in Ophthalmic and Vision Research and was approved by the Institutional Animal Care and Use Committee of the VA Boston Healthcare System.

### Animals and tissue preparation

P23H-line 1 homozygous rats of 17–45 weeks of age were used in this study. At this age range, rod photoreceptors are largely absent with only cone photoreceptors remaining [3]. Breeding pairs of P23H-line 1 homozygous rats were generously donated by Dr. Matthew LaVail (University of California San Francisco, CA). The room light was kept on a 12 hr light/dark cycle using standard fluorescent lighting. During the light cycle, the illumination at the level of the cages was 100–200 lux.

On the day of an experiment, a rat was euthanized with sodium pentobarbital (150 mg/kg, i.p.), and the eyes were removed and hemisected under normal room light. After removal of the vitreous humour from each eye, one eyecup was transferred to a holding vessel containing bicarbonate-buffered Ames medium (Sigma-Aldrich), which was continuously gassed at room temperature with 5% CO_2_/95% O_2_. The retina of the other eyecup was gently peeled from the retinal pigment epithelium/choroid and trimmed into a square of ∼12 mm^2^. The retina was then placed photoreceptor side down in a small-volume (0.1 ml) chamber. The chamber was mounted on a fixed-stage upright microscope (Nikon Eclipse E600FN), and the retina superfused at 1.5 ml/min with bicarbonate-buffered Ames medium supplemented with 2 mg/ml D-(+) glucose and equilibrated with 5% CO_2_/95% O_2_. An in-line heating device (Warner Instruments) was used to maintain recording temperature at 35–36°C. The retina of the other eyecup was used later in the day.

### Electrophysiology

In this study, action potentials (spikes) were recorded extracellularly from individual RGCs. With the aid of red light (>630 nm) that was delivered from below the chamber, the tip of a glass-insulated platinum/tungsten microelectrode (0.6–1.0 MΩ impedance; Thomas Recording GmbH, Germany) was visually advanced to the retinal surface with a motor-driven micromanipulator. Extracellular potentials from RGCs were amplified and bandpass filtered at 100 to 5000 Hz by a differential amplifier (Xcell-3; FHC, Bowdoin, ME). To ensure that recordings were made from single cells, the recorded waveform of the spike was continuously displayed in real time on a PC to check for uniformity of spike size and shape. Spikes from single RGCs were converted to standard transistor to transistor logic (TTL) pulses with a time-amplitude window discriminator (APM Neural Spike Discriminator, FHC). A laboratory data acquisition system (1401 Processor and Spike2 software; Cambridge Electronic Design Ltd., Cambridge, UK) was used to digitize the TTL pulses and raw spike train data.

### Light stimulation

Light from a mercury arc lamp illuminated an aperture that was focused on the retina from above, through the 4X objective of the microscope. The image produced on the retina was either a 250-µm or 1.5-mm diameter spot, which was centered on the recorded RGC. In the light path was a 545 nm interference filter (bandwidth, 30 nm). The intensity of the unattenuated light stimulus on the retina, measured with a spectroradiometer (ILT900-R, International Light Technologies), was 8.5×10^17^ photons/cm^2^/s. Neutral density filters were inserted in the light path to reduce the intensity of light stimulus. An electromechanical shutter (Uniblitz, Rochester, NY) was used to control the stimulus duration, which was set to 100 ms in constructing intensity-response curves. During recordings from RGCs, light flashes were presented with interstimulus intervals of 3–6 s to avoid any adapting effect of the previous flash. RGCs were classified as either ON-center or OFF-center from their response to a 500–700 ms flash of light (250-µm diameter spot). A long duration flash was used in order to avoid the possibility of misclassifying an ON-center cell with a long-latency response as an OFF-center cell. Occasionally, a cell was encountered that elicited both ON and OFF responses to a 250-µm diameter spot of light. These cells were excluded from this study. All experiments were performed in a dimly lighted room (10 lux).

### Drug preparation and delivery

The mGlu1 receptor antagonist JNJ16259685 (3,4-dihydro-2*H*-pyrano[2,3-*b*]quinolin-7-yl)-(*cis*-4-methoxycyclohexyl)-methanone), the GABA_A_ receptor antagonist SR95531 (6-imino-3-(4-methoxyphenyl)-1(6*H*)-p yridazinebutanoic acid hydrobromide), the GABA_C_ receptor agonist TACA (*trans*-4-aminocrotonic acid), and the GABA_C_ receptor antagonist TPMPA (1,2,5,6-tetrahydropyridin-4-yl) methylphosphinic acid) were purchased from Tocris Bioscience. JNJ16259685 was dissolved in DMSO and diluted at least 100-fold in isotonic saline solution. SR95531, TACA and TPMPA were dissolved in isotonic saline solution. The drug solutions were applied to the bathing solution at a steady rate via motorized syringe pumps (Razel Scientific Instruments). The final concentration of DMSO at the retina was ≤0.01%. (At this level, DMSO had no noticeable effects on RGC responses.) A drug was bath applied for ∼10 min to ensure stable responses before its effects were tested. Only one cell was studied in each retina to avoid possible residual drug effects.

### Data analysis

The light responses of RGCs were calculated by counting the number of spikes within a 100 ms window that encompassed the peak response and subtracting any baseline (spontaneous) activity, measured between light stimuli. Cell responses were averaged from 5 stimulus presentations. Intensity-response curves of RGCs were fitted with a sigmoidal dose-response (variable slope), using SigmaPlot 10.0 (SPSS, Chicago, IL). Three parameters were measured from the curve fits: maximum peak response, dynamic operating range, and light sensitivity. The maximum peak response was simply the result from the fit of data points. The dynamic operating range was defined as the range of light intensity that elicited responses between 10 and 90% of maximum peak response. Drug-induced change in light sensitivity was determined by comparing the light intensity that evoked a half-maximum response prior to drug application with the light intensity that evoked the same peak response in the presence of the drug. Data are expressed as the mean ± standard deviation. Statistical significance was assessed using paired Student's t-tests, with P<0.05 considered significant.

## Results

JNJ16259685 is a highly selective, potent mGlu1 receptor antagonist [18], [19]. I examined the effects of bath-applied JNJ16259685 (0.5 µM) on 33 P23H rat RGCs that were stimulated with either a 250-µm or 1.5-mm diameter spot of light centered over the receptive field. Nineteen RGCs were stimulated with the small spot of light; 14 RGCs were stimulated with the large spot of light. Since the effects of JNJ16259685 on the measured variables did not reveal significant differences, data from both spot stimuli were pooled in the overall analysis. Of the 33 RGCs, 20 were ON-center cells and 13 were OFF-center cells.

### Effect of JNJ16259685 on light sensitivity of P23H rat RGCs

**Figure 1:**
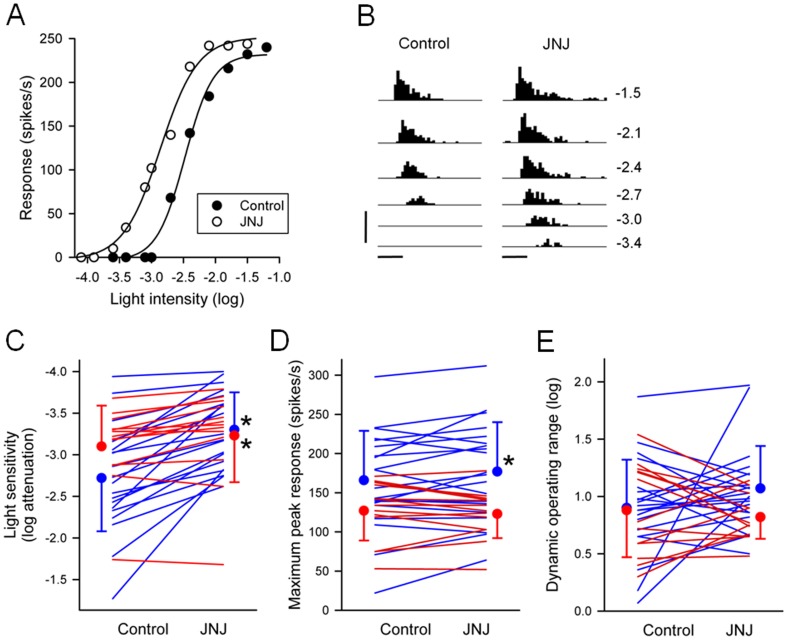
Effects of JNJ16259685 on light responses of P23H rat RGCs. (**A**) Intensity-response curves from an ON-center RGC, taken before and during application of JNJ16259685 (0.5 µM) to the bathing solution. The abscissa is labeled as log-unit attenuation in stimulus intensity from the maximum (8.5×10^17^ photons/cm^2^/s). (**B**) Post-stimulus time histograms (10-ms bin width) of spike responses from the same RGC at six different light intensities. The values on the right are the number of log units of attenuation. Timing and duration (100 ms) of the light stimuli are indicated by the horizontal bars at the bottom. Vertical bar, 500 spikes/s. JNJ16259685-induced change in (**C**) light sensitivity, (**D**) maximum peak response, and (**E**) dynamic operating range of P23H rat RGCs (n = 33). Individual ON-center RGCs (n = 20) and OFF-center RGCs (n = 13) are connected by blue and red lines, respectively. Each circle and vertical bar represents the mean and standard deviation of the mean. Asterisk indicates significantly different from control (P<0.05).

Figure 1A and B shows the effects of JNJ16259685 on an ON-center RGC. The light intensity that evoked a half-maximum response prior to application of JNJ16259685 was –2.48 log units attenuation. With application of JNJ16259685, the light intensity that evoked the same response was –2.92 log units attenuation. By definition, JNJ16259685 had increased the sensitivity of this cell to light by 0.44 log unit. JNJ16259685 increased the sensitivity of all 20 ON-center RGCs and 10 of the 13 OFF-center RGCs (Fig. 1C). For ON-center RGCs, the light intensity that generated a half-maximal response prior to application of JNJ16259685 was on average –2.72±0.64 log units attenuation. In the presence of JNJ16259685, the same light response was obtained at a light intensity of –3.30±0.45 log units attenuation (0.58 log unit lower intensity). The difference of the means was statistically significant (P<0.001; paired t-test). For OFF-center RGCs, the light intensity that generated a half-maximal response prior to application of JNJ16259685 was on average –3.10±0.49 log units attenuation. In the presence of JNJ16259685, the same light response was obtained at a light intensity of –3.23±0.56 log units attenuation (0.13 log unit lower intensity). The difference of the means was statistically significant (P = 0.007; paired t-test). I found that the effects of JNJ16259685 were long-lasting, with no recovery even 60 min after the onset of washout. Similar long-lasting effects of JNJ16259685 were reported by Fukunaga et al. [18] on EPSPs recorded from Purkinje cells in rat cerebellar slices. They attributed this to the lipophilicity of JNJ16259685.

**Figure 2:**
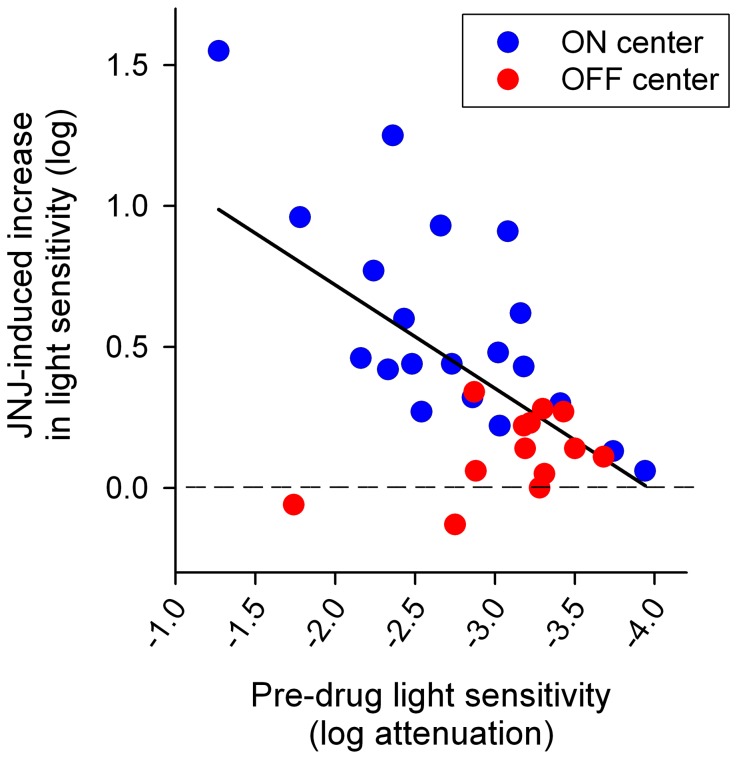
Comparison of JNJ16259685-induced increase in light sensitivity and pre-drug light sensitivity of P23H rat RGCs. Measurements from individual ON-center RGCs (n = 20) and OFF-center RGCs (n = 13) are represented by blue and red circles, respectively. The solid line indicates the linear regression fit of all data points. Pearson's correlation coefficient was 0.585 (P<0.001).

The effect of JNJ16259685 tended to be greater in RGCs that were initially less sensitive to light. This is illustrated in the scatter plot of Figure 2. A moderate correlation was found between the JNJ16259685-induced increase in light sensitivity and the pre-drug light sensitivity. Note that most OFF-center RGCs encountered in this study had a relatively high pre-drug light sensitivity. This could explain why OFF-center RGCs, as a group, were less affected by JNJ16259685.

### Effect of JNJ16259685 on maximum peak response of P23H rat RGCs

Figure 1A shows that JNJ16259685 increased the maximum peak response of the ON-center RGC from 232 to 252 spikes/s. JNJ16259685 increased the maximum peak response of 13 of the 20 ON-center P23H rat RGCs studied (Fig. 1D). On average, the maximum peak response prior to application of JNJ16259685 was 166±63 spikes/s. With application of JNJ16259685, the maximum peak response increased to 177±63 spikes/s. This 7% increase was statistically significant (P<0.046; paired t-test). For OFF-center RGCs (n = 13), the maximum peak response was 127±38 spikes/s prior to application of JNJ16259685 and 123±31 spikes/s in the presence of JNJ16259685. The difference in values was not statistically significant (P = 0.369; paired t-test).

### Effect of JNJ16259685 on dynamic operating range of P23H rat RGCs

Figure 1E shows the effect of JNJ16259685 on the dynamic operating range of ON-center and OFF-center RGCs. The dynamic operating range of ON-center RGCs averaged 0.90±0.42 log unit prior to application of JNJ16259685 and 1.07±0.37 log unit in the presence of JNJ16259685. This difference in the dynamic operating range was not statistically significant (P = 0.141; paired t-test). The dynamic operating range of OFF-center RGCs averaged 0.88±0.41 log unit prior to application of JNJ16259685 and 0.82±0.19 log unit in the presence of JNJ16259685. This difference in the dynamic operating range was not statistically significant (P = 0.518; paired t-test).

### Effects of SR95531 on light responses of P23H rat RGCs

Vigh et al. [15] have shown that activation of mGlu1 receptors in the goldfish retina leads to activation of both GABA_A_ and GABA_C_ receptors on goldfish bipolar cell axon terminals. Bipolar cell axon terminals in the rat retina also express GABA_A_ and GABA_C_ receptors [12], [13], [16], [20]. Although it is known that blocking GABA_C_ receptors increases the light responsiveness of RGCs in P23H rat retina [10], the effect of a GABA_A_ receptor antagonist on the light responsiveness of RGCs in the P23H rat retina has not yet been studied.

**Figure 3:**
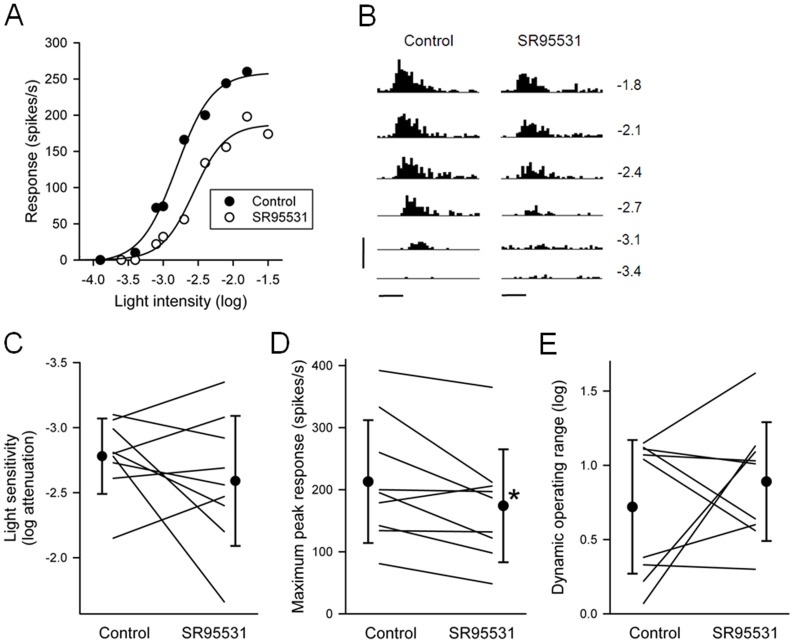
Effects of SR95531 on light responses of ON-center P23H rat RGCs. (**A**) Intensity-response curves from one RGC, taken before and during application of SR95531 (5 µM) to the bathing solution. The abscissa is labeled as log-unit attenuation in stimulus intensity from the maximum (8.5×10^17^ photons/cm^2^/s). (**B**) Post-stimulus time histograms (10-ms bin width) of spike responses from the same RGC at six different light intensities. The values on the right are the number of log units of attenuation. Timing and duration (100 ms) of the light stimuli are indicated by the horizontal bars at the bottom. Vertical bar, 500 spikes/s. SR95531-induced change in (**C**) light sensitivity, (**D**) maximum peak response, and (**E**) dynamic operating range of ON-center P23H rat RGCs. Individual RGCs (n = 9) are connected by lines. Each circle represents the mean (± standard deviation) value. Asterisk indicates significantly different from control (P<0.05).

Since the effects of JNJ16259685 on the light responses of RGCs were greater in ON-center cells than in OFF-center cells, I examined the effects of the GABA_A_ receptor antagonist SR95531 (5 µM) on the light responses of ON-center RGCs. Figure 3A and B shows the effect of SR95531 on an ON-center RGC. SR95531 decreased the light sensitivity of this cell by 0.41 log unit and decreased the maximum peak response by 28%. Following a 10 min washout of SR95531, at which time the light responses returned to near control levels (data not shown), the GABA_C_ receptor antagonist TPMPA was bath applied. As expected from a previous study [10], TPMPA increased the light sensitivity and maximum peak response of this cell. Figure 3C, D, and E summarizes the findings obtained with SR95531 on all ON-center RGCs (n = 9) studied. No statistically significant effect was found with SR95531 on either the light sensitivity (P = 0.296; paired t-test) or dynamic operating range (P = 0.389; paired t-test) of the RGCs. On average, SR95531 decreased the maximum peak response by 18% (from 213±99 to 174±91 spikes/s). This decrease in maximum peak response was statistically significant (P = 0.033; paired t-test).

### TPMPA occludes the effects of JNJ16259685 on P23H rat RGCs

**Figure 4:**
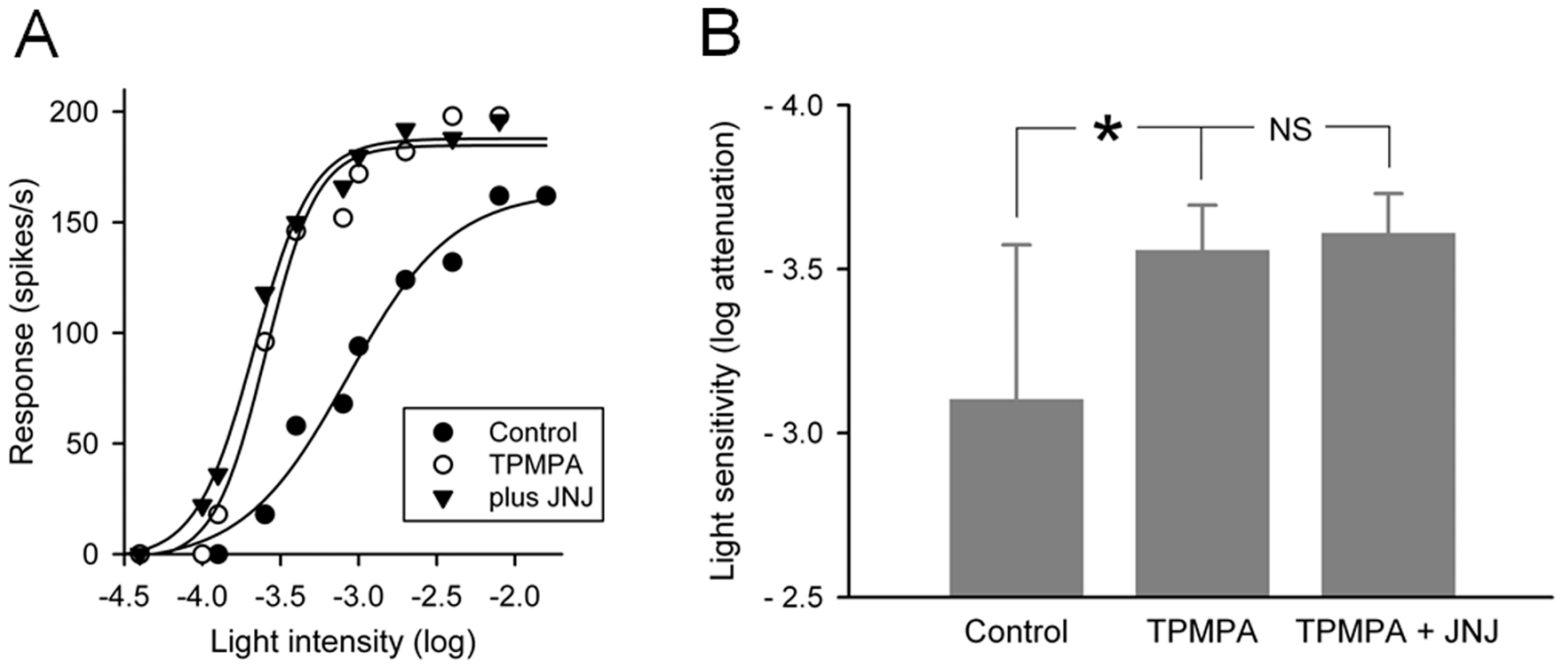
Occlusion of JNJ16259685 effect on light sensitivity of ON-center P23H rat RGCs by a GABA_C_ receptor antagonist. (**A**) Intensity-response curves from one RGC, taken before drug application, during application of TPMPA (100 µM), and after addition of JNJ16259685 (0.5 µM) to the bathing solution. The abscissa is labeled as log-unit attenuation in stimulus intensity from the maximum (8.5×10^17^ photons/cm^2^/s). (**B**) Summary of experimental results. Application of TPMPA (100 µM) causes a significant (* P<0.05) increase in light sensitivity compared to control. Addition of JNJ16259685 (0.5 µM) to the TPMPA-containing bathing solution had no statistically significant effect. Data are expressed as the mean and standard deviation of the mean (n = 6).

If JNJ16259685 is affecting P23H rat RGCs by reducing GABA release onto GABA_C_ receptors, then blocking GABA_C_ receptors with TPMPA should occlude the effects of JNJ16259685. I tested this hypothesis on ON-center RGCs, which as a group are affected by JNJ16259685 more so than OFF-center RGCs, by applying this mGlu1 receptor antagonist in the presence of TPMPA. Figure 4A shows findings obtained from one ON-center RGC. Before application of any drug, the light intensity that generated a half-maximal response was –3.07 log units attenuation. When TPMPA (100 µM) was applied to the retina, the RGC became more sensitivity to light. The light intensity that generated a half-maximal response was now –3.64 log units attenuation. In the presence of TPMPA, JNJ16259685 (0.5 µM) was applied to the retina. Light sensitivity was essentially unchanged, only a 0.06 log unit increase. Figure 4B summarizes the findings from all ON-center P23H rat RGCs (n = 6) examined. On average, TPMPA increased light sensitivity of ON-center RGCs by 0.45 log unit. In the presence of TPMPA, JNJ16259685 caused only a slight (0.05 log unit) increase in light sensitivity, which was not found to be statistically significant (P = 0.129; paired t-test). Furthermore, in the presence of TPMPA, JNJ16259685 had no statistically significant effect (P = 0.286; paired t-test) on the maximum peak response of these RGCs either (data not shown). In conclusion, these results are consistent with the hypothesis that the effects of JNJ16259685 on RGCs are mediated through a decrease in activation of GABA_C_ receptors.

### TACA tends to reverse the effects of JNJ16259685 on P23H rat RGCs

I examined whether the GABA_C_ receptor agonist TACA could reverse the effects of JNJ16259685 on ON-center RGCs. In the presence of JNJ16259685 (0.5 µM), TACA (10–30 µM) decreased the light sensitivity of 5 of eight ON-center RGCs tested. For the five ON-center RGCs, light sensitivity decreased on average by 0.28 log unit (from –3.28±0.65 to –3.00±0.71 log units). The light sensitivity of the other 3 RGCs remained unchanged. One possible explanation for the lack of an effect is that TACA is a potent competitive inhibitor of GABA uptake [21]. If TACA should serve as a substrate for GABA transporters, then this would be expected to limit the concentrations of TACA achieved at synaptic receptors.

### Effects of JNJ16259685 on light responses of SD rat RGCs

**Figure 5:**
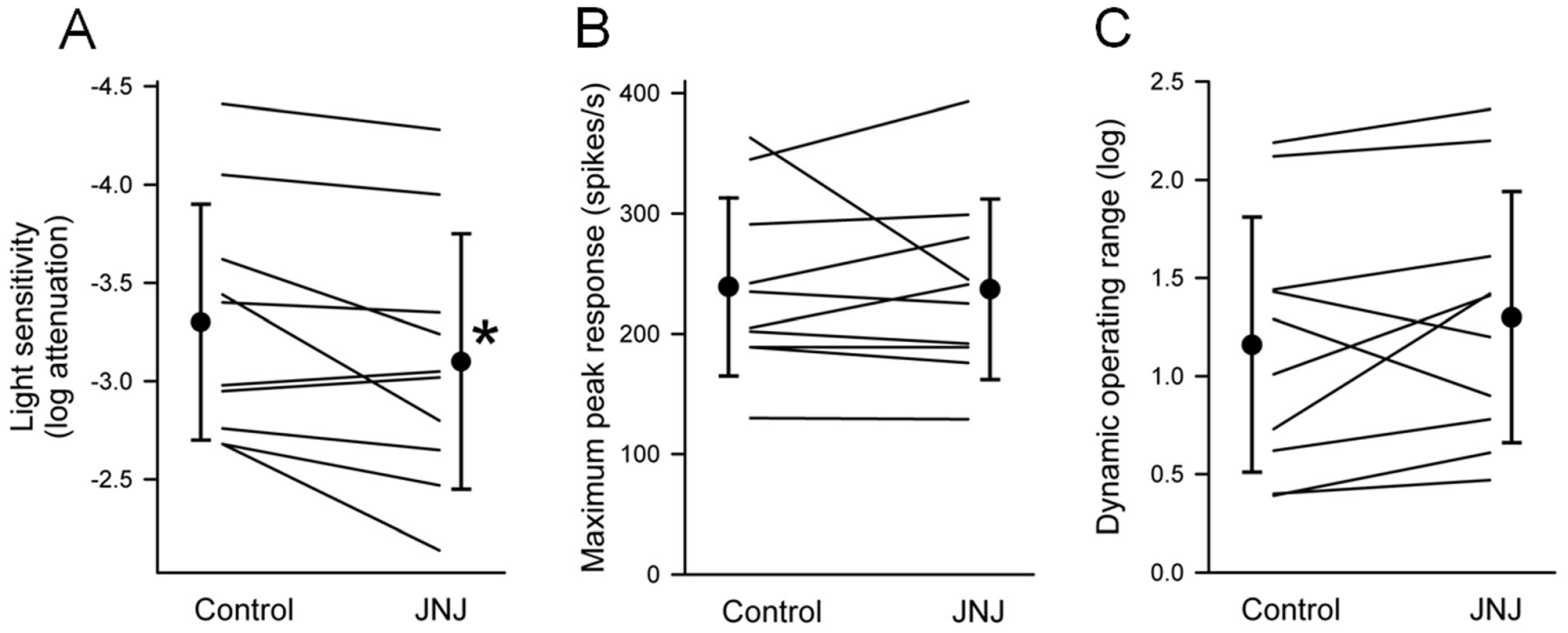
Effects of JNJ16259685 on light responses of ON-center SD rat RGCs. JNJ16259685-induced change in (**A**) light sensitivity, (**B**) maximum peak response, and (**C**) dynamic operating range of SD rat RGCs. Individual RGCs (n = 10) are connected by lines. Each circle represents the mean (± standard deviation) value. Asterisk indicates significantly different from control (P<0.05).

Previously, I reported that TPMPA decreased light sensitivity of ON-center SD rat RGCs (on average by 0.20 log unit) but had no statistically significant effect on either the maximum peak response or dynamic operating range. I examined the effects of JNJ16259685 on the light responses of ten ON-center SD rat RGCs (4 cells were stimulated with a small spot of light; 6 cells with a large spot). Similar to what I observed with TPMPA, I found that JNJ16259685 decreased light sensitivity of these cells but had no statistically significant effect on either the maximal peak response or dynamic operating range (Fig. 5). The light intensity that generated a half-maximal response prior to application of JNJ16259685 was on average –3.30±0.60 log units attenuation. In the presence of JNJ16259685, the same light response was obtained at a light intensity of –3.10±0.65 log units attenuation. The difference of the means was statistically significant (P < 0.028; paired t-test). Thus, like TPMPA, JNJ16259685 decreased light sensitivity of ON-center SD rat RGCs by 0.20 log unit.

## Discussion

The principal findings of this study are that blocking mGlu1 receptors in the retinas of P23H transgenic rats increases light sensitivity of ON-center RGCs and (to a lesser extent) OFF-center RGCs, and increases the maximum peak response of ON-center RGCs. Furthermore, I found that the effects of the mGlu1 receptor antagonist JNJ16259685 on ON-center RGCs are occluded by the GABA_C_ receptor antagonist TPMPA, which is consistent with the hypothesis that the effects of JNJ16259685 on RGCs are mediated through a decrease in activation of GABA_C_ receptors.

### Previous studies on retinal mGlu1 receptors

In the rat retina, the presence of mGlu1 was first shown with in situ hybridization at the mRNA level [22]. Both amacrine cells and RGCs were labeled for the mGlu1 receptor mRNA. Labeling was not found in either bipolar cells or Müller glial cells. Immunostaining and electron microscopic studies in the rat retina have shown that mGlu1 receptors are present in the dendrites of rod bipolar cells and in amacrine cell processes postsynaptic to axon terminals of OFF-cone, ON-cone and rod bipolar cells [17]. From their material they were not able to say whether ganglion cell dendrites were also labeled for mGlu1 receptors, although in an earlier study [23] a few large ganglion cell bodies in the rat retina were found to be immunostained for mGlu1 receptors. In the cat retina [24], the immunostaining pattern for mGlu1 receptors is similar to what Koulen et al. [17] observed in the rat retina, with two notable exceptions: 1) the synaptic terminals of rod photoreceptors but not the dendrites of rod bipolar cells were labeled, and 2) no labeling of amacrine cell processes postsynaptic to axon terminals of rod bipolar cells were observed.

Several physiological studies have examined the function of mGlu1 receptors in the retina. From whole-cell recordings of Mb-type bipolar cell terminals in the goldfish retina, Vigh et al. [15] found that activation of mGlu1 receptors in the retina stimulates GABA release from amacrine cells onto GABA receptors on the bipolar cell terminals. An earlier study by Vigh and Lasater [25] showed that activation of mGlu1 receptors on teleost GABAergic amacrine cells decreases GABA_A_ responses in these cells. This is one possible mechanism for the mGlu1 receptor-mediated increase in GABA release from the amacrine cells in the goldfish retina. Another possibility, suggested by Vigh et al. [15], is that mGlu1 receptor activation increases the release probability of GABA release directly through an increase in intracellular Ca^2+^ levels. More recently, Yu et al. [26] found that in a rat RGC-astroglia coculture system, activation of mGlu1 receptors depolarizes RGCs by blocking a background K^+^ conductance in these cells. Although undoubtedly there are other yet to be discovered functional roles for mGlu1 receptors in the retina, the effects of the mGlu1 receptor antagonist JNJ16259685 observed in the present study can largely be explained by the modulation of GABA release from amacrine cells onto bipolar cells.

### Comparison with the GABA_C_ receptor antagonist TPMPA

I found that the mGlu1 receptor antagonist JNJ16259685 caused a 3.8-fold increase in light sensitivity of ON-center RGCs and a 1.3-fold increase in light sensitivity of OFF-center RGCs. These findings are similar to the findings obtained with the GABA_C_ receptor antagonist TPMPA [10], which increased the light sensitivity of ON-center RGCs by 4.3-fold and OFF-center RGCs by 2.4-fold. The increase in light sensitivity of ON-center RGCs by the two receptor antagonists is quantitatively very similar. The effect of JNJ16259685 on the light sensitivity of OFF-center RGCs is less than that observed with TPMPA. However, since only 6 OFF-center RGCs were examined with TPMPA, this difference in effect magnitude will need to be reexamined in a larger sample size. Like TPMPA, JNJ16259685 increased the maximum peak response of ON-center RGCs and had no statistically significant effect on the maximum peak response of OFF-center RGCs. Furthermore, like TPMPA, JNJ16259685 had no statistically significant effect on dynamic operating range of either ON- or OFF-center RGCs. These findings, together with the finding that TPMPA occludes the effects of JNJ16259685 on ON-center rat RGCs, indicate that the effects of the mGluR1 receptor antagonist JNJ16259685 are mediated through a reduction in release of GABA onto GABA_C_ receptors.

### Effect of a GABA_A_ receptor antagonist on light responses

Activation of mGlu1 receptors potentiates both GABA_C_- and GABA_A_-mediated responses in goldfish bipolar cells [15]. If this should also occur in the rat retina, then blocking mGlu1 receptors would diminish both GABA_C_- and GABA_A_-mediated inhibition in bipolar cells. I found that blocking retinal GABA_A_ receptors with the GABA_A_ receptor antagonist SR95531 did not increase light responsiveness of ON-center RGCs. On the contrary, SR95531 decreases the maximum peak response of these RGCs. The cellular mechanism(s) for this decrease in maximum peak response is not clear. Unlike GABA_C_ receptors, which are located primarily on retinal bipolar cells [12], [13], GABA_A_ receptors are ubiquitous, present on bipolar cells, amacrine cells and RGCs [27], [28]. To complicate matters, reducing GABAergic inhibition to GABAergic amacrine cells leads to an increase in GABA input to bipolar cells, thereby increasing stimulation of GABA_C_ receptors. This has been shown to occur in both goldfish [15] and mouse [29] retinas.

## Concluding Remarks

At the ages of the P23H rats used in this study, only remnant, cone photoreceptors remain [3]. The results of this study indicate that blocking retinal mGlu1 receptors in this rat model of RP potentiates transmission of any, weak signals originating from the cone photoreceptors. Presumably this augmentation of photoreceptor-mediated signals to RGCs occurs through a reduction in GABA_C_-mediated activity on axon terminals of bipolar cells.
